# SDR Proof-of-Concept of Full-Duplex Jamming for Enhanced Physical Layer Security

**DOI:** 10.3390/s21030856

**Published:** 2021-01-28

**Authors:** André Silva, Marco Gomes, João P. Vilela, Willie K. Harrison

**Affiliations:** 1 Department of Informatics Engineering, Instituto de Telecomunicações, University of Coimbra, CISUC, 3030-290 Coimbra, Portugal; uc2015228086@student.uc.pt; 2 Department of Electrical and Computer Engineering, Instituto de Telecomunicações, University of Coimbra, 3030-290 Coimbra, Portugal; 3 CRACS/INESCTEC, CISUC and Department of Computer Science, Faculty of Sciences, University of Porto, 4169-007 Porto, Portugal; jvilela@fc.up.pt; 4 Department of Electrical and Computer Engineering, Brigham Young University, Provo, UT 84602, USA; willie.harrison@byu.edu

**Keywords:** physical-layer security, jamming, full duplex, self-interference cancellation, software-defined radio

## Abstract

In order to secure wireless communications, we consider the usage of physical-layer security (PLS) mechanisms (i.e., coding for secrecy mechanisms) combined with self-interference generation. We present a prototype implementation of a scrambled coding for secrecy mechanisms with interference generation by the legitimate receiver and the cancellation of the effect of self-interference (SI). Regarding the SI cancellation, four state-of-the-art algorithms were considered: Least mean square (LMS), normalized least mean square (NLMS), recursive least squares (RLS) and QR decomposition recursive least squares (QRDRLS). The prototype implementation is performed in real-world software-defined radio (SDR) devices using GNU-Radio, showing that the LMS outperforms all other algorithms considered (NLMS, RLS and QRDRLS), being the best choice to use in this situation (SI cancellation). It was also shown that it is possible to secure communication using only noise generation by the legitimate receiver, though a variation of the packet loss rate (PLR) and the bit error rate (BER) gaps is observed when moving from the fairest to an advantageous or a disadvantageous scenario. Finally, when noise generation was combined with the adapted scrambled coding for secrecy with a hidden key scheme, a noteworthy security improvement was observed resulting in an increased BER for Eve with minor interference to Bob.

## 1. Introduction

Secrecy against eavesdroppers has always been a concern in communication systems. This threat is particularly relevant in wireless communications where, due to their broadcast nature, it is difficult to restrict the communication to an intended receiver (Bob) while guaranteeing secrecy against an illegitimate one (Eve) [1]. 5G and internet-of-things (IoT), with “everything” connected, have just aggravated these concerns [2].

Shannon was the first to address the secrecy problem [3] paving the way for the study of information theoretically secure coding schemes. However, practical schemes able to guarantee information theoretic security are still unknown for real world channels [4]. Instead, security and secrecy of today’s communications mainly rely on modern cryptographic schemes that settle on the premise of the computational infeasibility of breaking the cryptosystem [5]. However, the definition of infeasible changes over time as technology matures. Besides, new vulnerabilities are always being found in these schemes compromising the security; for instance, the “Heartbleed” bug [6] was a serious vulnerability in OpenSSL cryptographic software library compromising all communications using Secure Socket Layer (SSL)/Transport Layer Security (TLS) over the Internet. In addition, the traditional encryption-based algorithms and standards are not always perfectly appropriate for resource-limited IoT scenarios because of the computational cost and energy consumption [7].

Physical layer security (PLS) has found renewed interest recently as an additional means of defence to realise wireless secrecy in communications [8,9]. PLS exploits the random characteristics of the medium, such as noise, fading, and interference, along with the several degrees of freedom granted on the design of a communication system such as coding design, MIMO capability, beamforming, relaying, cooperative communications, jamming, etc. [9,10]. PLS can prove useful as a complement to modern cryptography to further secure wireless communications without relying on the computational intractability of certain functions. Also, it should be emphasized that this level of security is placed as a complement and not a replacement to modern cryptography.

**Figure 1:**
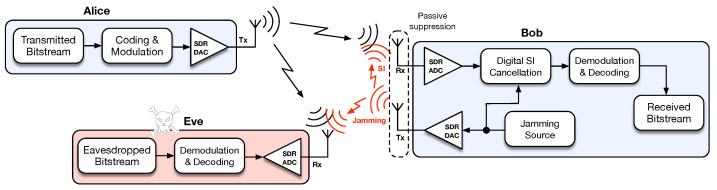
System setup for implementing self-interference cancellation for secrecy.

The origin of PLS traces back, in fact, to Shannon [3] and Wyner [11], although with a surge of interest in latter years. Wyner in his seminal work [11] defined the concept of the wiretap channel that considers a three-party communication setup, composed of a legitimate transmitter and receiver (Alice and Bob) and an eavesdropper (Eve), and in which the wiretap channel between Alice and Eve is degraded with respect to the main channel between Alice and Bob. Also, under this setup, Wyner showed that it is possible to build wiretap codes to achieve reliable communication to Bob and secrecy against Eve. Motivated by this result several practical coding schemes have been proposed [8,12,13]. However, most of these schemes rely on Wyner’s wiretap channel assumption of Eve having a disadvantage with respect to Bob, which is difficult to assure at all times. This led to the development of friendly/cooperative jamming schemes where there is one helper node causing interference to degrade Eve’s channel [14,15,16]. However, these type of schemes have drawbacks such as the need for synchronization and willingness to cooperate by the helper node. This work addresses these drawbacks through a solution in which Bob (rather than a third party) acts himself as a jammer as shown in Figure 1, while transmitting in the same frequency in which he is receiving (i.e., full-duplex (FD)), therefore suffering from self-interference (SI) upon reception [17]. Although there are already works on FD jamming for secrecy purposes [18,19], as far as we are aware there is no practical proof-of-concept with a hardware/software implementation (the authors would like to disclose that this work has been presented, only in part, at the international conference CSNDSP2020 [20]). Rather, existing works with implementations of physical-layer security schemes tend to focus on secret-key agreement strategies [21,22], deriving security from signaling efforts such as beamforming, channel estimation and spread spectrum [23,24,25], or using the physical layer to authenticate users through radio signal analysis [26,27,28].

In this work we combine coding for secrecy mechanisms with FD interference generation by the legitimate receiver and the cancellation of the effect of SI, and we make a proof-of-concept of this scheme showing it to be possible to secure communication using only noise generated by Bob, even when Bob has a degraded channel with respect to Eve. We consider the use of interleaved/scrambled coding for secrecy with a hidden key (ICS-HK/SCS-HK) [29,30]. This state-of-the-art coding mechanism is a three stage coding scheme [4] whereby a random key that is used to scramble/interleave data [31,32] is punctured before transmission to produce a so called cliff-effect of forward error-correcting codes [33]. Secrecy can be achieved as long as Eve experiences a worse signal-to-interference-plus-noise ratio (SINR) upon reception as compared to Bob. This is assured by the use of FD jamming and SI cancellation by Bob. Regarding the SI cancellation, four state-of-the-art algorithms are considered: least mean square (LMS), normalized least mean square (NLMS), recursive least squares (RLS) and QR decomposition recursive least squares (QRDRLS) [34,35]. In summary, this paper contributes to the literature by providing a software-defined radio (SDR) implementation of the aforementioned scheme using Ettus Universal Software Radio Peripheral (USRP) B210 devices, and GNU radio [36]. The paper further provides an evaluation in a real-world scenario, and a critical analysis on the performance of SCS-HK added by Bob’s FD jamming, and the effectiveness of several SI cancellation algorithms that can be employed by Bob.

The remainder of the paper is organized as follows. In Section 2, concepts of PLS are introduced along with the mechanisms used on the prototype implementation, which is described in Section 3. In Section 4 the setups and metrics are explained and in Section 5 the results are presented. Finally, Section 6 concludes the paper.

## 2. Background

In this chapter we introduce the SCS-HK coding and the FD jamming schemes, which are the basis for this work. We highlight the role of jamming for security, and address FD concepts along with its main disadvantage of self-interference, and how it can be canceled. We also address practical issues such as the criteria for selection of the jamming power level, the energy cost of jamming, and the decoding strategies that can be used by Eve to combat the jamming and attempt to retrieve the information.

### 2.1. SCS-HK Adapted Scheme

The ICS-HK and SCS-HK coding schemes [29,30] rely on interleaving/scrambling the information with a random key, followed by encoding them together with an LDPC code. Some of the bits of the resultant codeword are punctured before the transmission to guarantee that only the legitimate receiver having a better channel can correctly recover the message. As shown in [37] adaptive PLS may be achieved with the ICS-HK and SCS-HK techniques by adjusting the number of punctured bits according to the channel conditions between Alice and Bob, assuming the existence of a feedback channel through which Bob can inform Alice of his reception conditions.

The SCS-HK scheme [29] was adapted to be used in this prototype, where the modifications were mainly in the type of scrambler used (additive rather than multiplicative) and how a packet is found (a simple verification after the header being decoded through a Reed-Solomon code). The first modification was performed to tackle the self-synchronizer capability of the multiplicative scrambler which grants information retrieval even without the correct seed/key. The latter was to maximize the throughput by removing the correlation operation which is computationally heavy and replace it with a bit-level comparison with a known word after being recovered with an error correction code.

**Figure 2:**
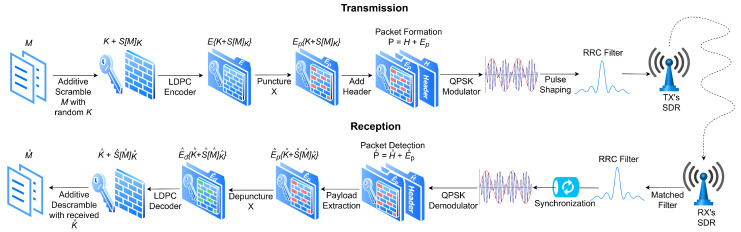
Adapted scrambled coding for secrecy with a hidden key (SCS-HK) scheme.

The SCS-HK adapted scheme architecture is represented in Figure 2. Firstly, the desired binary data for transmission *M* is scrambled using the additive scrambling (because it is not self-synchronizing, thus it is required to have the correct key to descramble it, or all the frame is lost) with a random key *K* resulting in $S{\left[M\right]}_{K}$. This scrambled data is then appended to *K* resulting in $K+S{\left[M\right]}_{K}$ and then encoded using the LDPC encoder $E\{K+S{\left[M\right]}_{K}\}$ which results in the packet’s payload $E_{p}$ punctured with a pattern *X*. Then, a header *H* with a known sequence is added, so that it can be recognized on the receiver side to know that the incoming payload results into a packet formed by $P=H+E_{p}$. Finally, data is modulated with quadrature phase shift keying (QPSK) and pulse shaping is performed by applying the root raised cosine filter, before being transmitted with the SDR. Through the use of scrambling and puncturing of the key, this scheme aims to increase the degradation of the wiretap channel to Eve, while causing limited impact on the legitimate channel to Bob.

### 2.2. Jamming and Cooperative Jamming

In the coding for secrecy schemes it is necessary for Bob to have some advantage over Eve, often an uncontrollable factor. Cooperative jamming [14,15,16] was considered to overcome this challenge to help lower the signal-to-noise ratio (SNR) of the wiretap channel. It consists of having an external jammer to help Alice to communicate with Bob using several known schemes, namely cooperative jamming by Gaussian noise, by structured codes, and by alignment.

All these schemes have some disadvantages, for instance: (1) the synchronization, that is, the helper needs to be in sync with Alice to correctly help Bob to have the necessary advantage; (2) the willingness of the helper to spend his own energy in order to emit the jamming signal to make other communications secure; and (3) there has to be a trusted relationship between the helper and Bob, because if the helper stops jamming, then Eve can retrieve the signal. Using Bob as a jammer can overcome most of these issues since it helps to solve the synchronization problem, the issue of the need for cooperation and it facilitates the removal of interference (the receiving device knows the characteristics of the interference being generated).

### 2.3. Full-Duplex and Self-Interference

Full-duplex enables simultaneous uplink and downlink communication in the same frequency at the same time [38] which is useful in this context: Bob receives the transmitted signal and at the same time emits the jamming signal. The main drawback in FD is the self-interference that is generated due to the signal created on the transmitter antenna being fed back into the receiver antenna of the same device. On top of that, the SI signal has higher gain than the intended one. Thus, Bob will not only receive the information signal but also receive the jamming signal (with higher gain) that is generated resulting in a lower SINR of the channel [15].

Furthermore, to successfully retrieve the signal of interest it is required to reduce the SI signal to decode it correctly, so the goal of SI cancellation is to predict and model the distortions and compensate for them at the receiver antennas.

### 2.4. Self-Interference Cancellation

There are three different categories of SI cancellation mechanisms [38], namely passive suppression, analog cancellation and finally digital cancellation. Passive suppression is based on attenuating the SI signal by physically separating/changing somehow the transmitter antenna and the receiver antenna of the device. The analog SI cancellation is performed before the signal goes into the analog-to-digital converter (ADC) (e.g., by using a noise canceling integrated circuit). Digital SI cancellation works after the signal goes into the ADC and takes advantage of the knowledge of the interfering signal for the purpose of canceling it, and will be the focus of our work.

There are adaptive SI cancellation algorithms which iteratively adjust the coefficients of the signal in a way to predict the desired one. The least mean square (LMS), normalized least mean square (NLMS), recursive least squares (RLS) and QR decomposition recursive least squares (QRD-RLS) are state-of-the-art examples of such [34,35,39]. These algorithms operate with a given target/reference signal d(n) and the input signal x(n). The filter transfer function H(z) outputs y(n), which considers the statistics of x(n) and d(n) in order for the output signal y(n) to approximate the target/reference signal d(n). That is, after x(n) is filtered, y(n) is produced, which is an estimate of the target signal d(n) with some error of e(n).

The LMS algorithm [34,39] is based on the concept of the adaptive finite impulse filter (FIR) Wiener filtering, however, instead of using auto-correlation and cross-correlation operations to estimate the signal, it uses the steepest descent method to estimate the gradient vector and constantly update the filter taps’ weights to converge to the optimum filter weight vector while minimizing the mean square error (to converge to the minimum mean squared error).

The NLMS [39] is a version slightly modified of the LMS which corrects the gradient noise amplification problem caused by the weight’s adjustment being directly proportional to the input x(n).

While LMS aims to reduce the mean squared error between the estimated signal and the desired one, RLS tries to recursively find the taps’ weights using the least squares method [35,39]. Therefore, the LMS input signal needs to be stochastic, while the RLS works better for a deterministic environment (i.e., there is no randomness on the input signal or the input signal has slowly time-varying randomness).

QRD-RLS is based on the previous one, but uses the QR decomposition [35], to explore the good numerical properties (raised when the algorithms are numerically implemented, i.e., due to inaccuracy of the ADC), thus achieving a numerically more stable algorithm.

Finally, since the self-interference is canceled prior to decoding, Alice and Bob are free to use any selection of waveforms or modulation, including multi-carrier waveforms such as OFDM.

### 2.5. Full-Duplex Jamming Practical Issues

Although SI cancellation techniques may be employed by Bob in order to improve his SINR, there are several questions that may arise regarding the practical deployment of the FD jamming scheme that are addressed here.

#### 2.5.1. Control of Bob’s Jamming Power Level

There are two constraints that dictate the power level of Bob’s jamming signal. The first is comprised of the limits on jamming peak power and duration imposed by Bob’s power supply capability. When finite constraints must be applied, Bob is free to jam at a power level that matches his constraints. When the constraint on Bob’s power consumption is not strict, another aspect of the communication system can help to guide this decision. At the physical layer the main goal is to assure reliable communication between Alice and Bob, with PLS schemes working as an “add-on” layer to increase security against eavesdroppers while not compromising communication between Alice and Bob. In the scheme of Figure 1, reliable communication between Alice and Bob is a function of the SINR experienced by Bob, which is affected by his capacity to cancel the self-interference caused by his own jamming signal. Therefore, a simple procedure to be used by Bob while jamming is to reduce the jamming power to a level that bounds the signal quality at a level that guarantees successful decoding by Bob.

#### 2.5.2. Energy Cost of Jamming

The energy cost is one of the main drawbacks of employing jamming techniques for physical-layer security (PLS), and one of the reasons why third parties may not be willing to participate as jammers for the benefit of others. This motivates our setup, whereby the receiver Bob acts himself as a jammer for his own security benefit, hence avoiding the need for jamming by third parties. However, this energy cost may still be problematic, particularly for power constrained devices. To reduce this cost, signalling mechanisms such as that proposed in [15] can be employed to coordinate Bob’s jamming with the transmission from Alice, thus enabling Bob to jam only when necessary.

#### 2.5.3. Decoding Strategies for Eve

If Bob transmits a time-correlated jamming signal, it may be possible for Eve to learn the signal, and then attempt to remove it prior to decoding. The use of signalling mechanisms for coordinated jamming [15] (as previously referred) can prevent Eve from capturing a clean version of the jamming signal. The use of independent additive white Gaussian noise as the jamming source further prevents Eve from being able to gain useful information that would allow here to make use of adaptive interference algorithms.

In this work, we assume the jamming signal generated/transmitted by Bob is a white Gaussian noise signal (with uncorrelated samples) in the transmission band. For this reason, any receiver in the vicinity of Bob (including himself) must treat the jamming as an additional source of AWGN noise. Only Bob is therefore capable of interference cancellation techniques. An optimal strategy by the eavesdropper is then to simply maximize the received signal-to-noise ratio (SNR) by way of matched filtering.

## 3. SDR Testbed Implementation

In this work we explore the usage of the adapted SCS-HK scheme (Section 2.1) along with interference generation (Section 2.2) by the receiver which causes SI (Section 2.3) that must be canceled using the mechanisms described in Section 2.4. The schema of the proposed mechanisms is shown in Figure 1, whereby Bob generates jamming noise to be mixed with the transmitted signal in such a way that Eve cannot recover the signal of interest, while Bob uses the advantage of the knowledge of the generated jamming noise to better estimate the transmitted signal. The developed proof-of-concept testbed uses Ettus Universal Software Radio Peripheral (USRP) B210 devices, programmed using GNU radio (source code is available at [36]).

**Figure 3:**
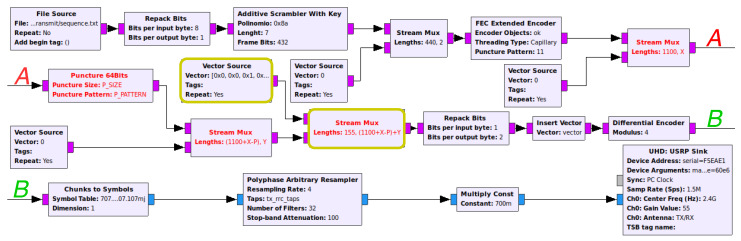
Alice’s flow graph of the real-world prototype.

Figure 3 represents a more detailed flow graph implementation of Alice’s side, which includes the SCS-HK scheme discussed. The data is obtained with the File Source block that creates samples that are adapted to be further managed by the Repack Bits block. The generated samples go through the Additive Scrambler with Key custom block to improve security (using the key as the seed) and enable symbol synchronization on the receiver side (to solve the whitening sequences problem). In the GNU-radio additive scrambler stock block, the input is treated as a continuous stream, i.e., without any seed reset; therefore, one bit error leads to multiple bit errors on the remaining stream. This can be solved by packetizing the output data of the scrambler, resetting the seed with a pre-established one and a frame of bits. Additionally, the SCS-HK uses a random key as the seed which is appended to the scrambled data. The scrambled data is then encoded using the FEC Encoder block with the algorithm that is defined in it, e.g., a (1100, 442) LDPC code in this case. The puncture component is the final step in which some bits of the payload are erased with a pre-established pattern $P_{\mathrm{PATTERN}}$. This operation is performed by the Puncture 64Bits custom block. Finally, the header is mixed with the payload by combining the Vector Source and the Stream Mux blocks, thus creating the final packet.

**Figure 4:**
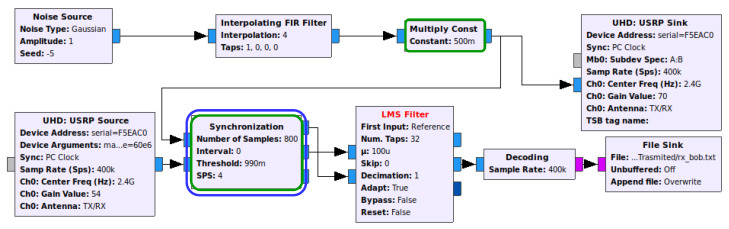
Bob’s flow graph of the real-world prototype generating noise and perform self-interference (SI) cancellation.

Figure 4 depicts Bob’s implementation, where the Decoding block is an aggregation of the necessary blocks to decode the signal transmitted from Alice, whereby a SI cancellation mechanism (e.g., LMS Filter) is employed. Finally, note that Eve’s flow graph is simply an attempt to decode the received signal with the Decoding block alone, without any knowledge of the interference generated by Bob.

The biggest challenge we faced when using the LMS/NLMS/RLS/QRD-RLS Filter blocks was that the noisy jamming sample that is mixed with the signal to be input in the second entry (i.e., picked up by Bob’s receiving antenna) must be time aligned with its respective original jamming sample, i.e., both inputs of the adaptive filter need to be synchronized (in regards to the jamming samples) in order to work properly. The problem is created because the path through USRP Sink block, air/wireless channel and USRP Source block takes more time to travel compared with the direct path on the flow graph. The solution found was to create a block that delays the noise stream to synchronize it with the receiving stream. Therefore, the block selects the first *X* noise samples and performs a correlation operation against the mixed signal to find the value needed to delay the noise stream. This block was called Synchronization and works with a sliding window with size *X* on top of the mixed stream to perform a correlation operation on that window. If the result of the correlation is greater than a desired threshold, the delay is found, and the noise stream is delayed by the determined value to align with the other stream. Otherwise, the window will slide and perform the correlation operation again, until convergence.

## 4. Setup and Metrics

Three different configurations were created to evaluate the SI cancellation capability of the adaptive algorithm to be chosen and the secrecy of information achieved.

**Figure 5:**
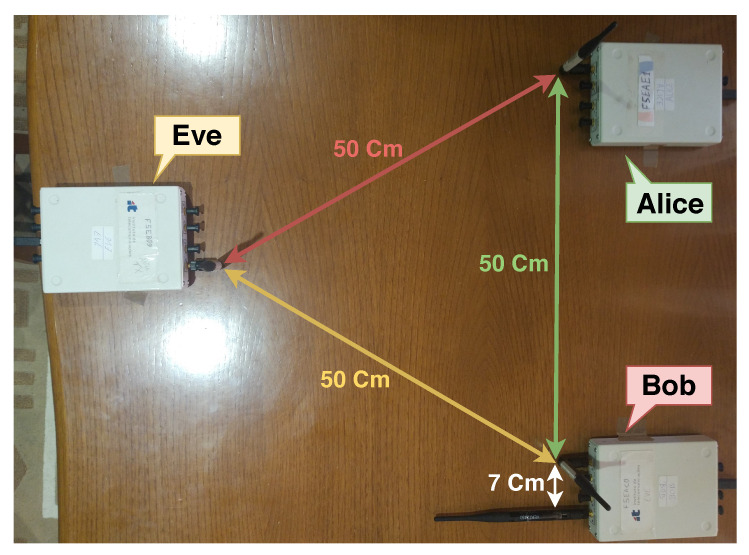
Setup I—Fairest setup possible.

The first setup (Setup I) recreates the fairest possible scenario, where all SDRs are evenly separated as illustrated in Figure 5. In this scenario, the signal power from Alice to Bob equals the signal power to Eve, thus, Bob needs to generate a noise signal which can superimpose the information signal on Eve, and at the same time cancel the SI.

**Figure 6:**
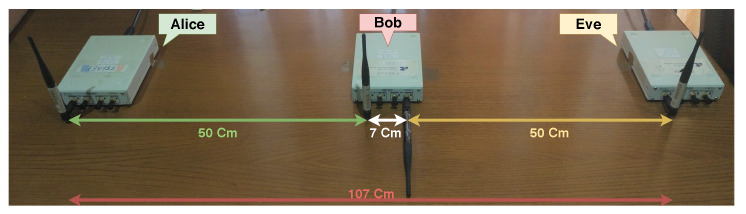
Setup II—Setup advantageous to Bob.

Figure 6 depicts Setup II, where Bob has an advantage because the distance between Alice and Bob is half of the Alice-Eve distance. This allows for Bob to receive a better signal from Alice than Eve; hence, Eve has a SNR significantly lower than Bob, which will allow degradation of the information signal with the same jamming power without affecting the SI capability of Bob.

**Figure 7:**
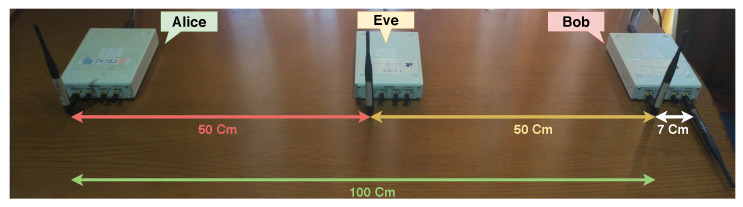
Setup III—Setup advantageous to Eve.

Finally, Setup III explores a disadvantageous scenario for Bob, where his distance to Alice is twice that of Eve’s distance, as shown on Figure 7. Therefore, Bob is receiving lower signal power from Alice than Eve, which will pressure Bob to decrease his jamming signal, otherwise he will not be able to recover the signal, and consequently, Eve will receive Alice’s signal with higher power.

To assess reception quality we will measure the error vector magnitude (EVM), i.e., the distance between the received constellation symbols and the ideal ones. A smaller EVM value, means better approximation to the constellation points, thus, better possibilities to correctly retrieve the information. In packet-based transmissions it is also possible to use as a metric the packet loss rate (PLR) and the bit error rate (BER). A packet loss occurs when some packet fails to successfully reach its destination in a network communication, either caused by errors in the transmission channel or by network congestion. The PLR is a practical metric that measures the percentage of lost packets relative to the total packets sent. Finally, the BER takes into account only the bits of successfully received packets at the output of the decoder and compares them with the original ones of the transmitted message. All of these metrics can be used in practical scenarios to evaluate security by stipulating reliability (for Bob) and secrecy (for Eve) levels based on each of these metrics, and computing the difference (i.e., gap) between the SNRs for which Bob and Eve achieve them.

Furthermore for statistically relevant results, in each evaluation and for each considered jamming power value, we perform 30 transmissions of $3.2\times 10^{6}$ bits each, for which the used metrics’ mean and 95% confidence interval were calculated.

## 5. Evaluation

In this section, we evaluate our combined SCS-HK and SI cancellation solution. Due to the large combination of possible setups and number of tests for each, we proceed as follows. We start by evaluating all SI cancellation algorithms in the fairest setup (Setup I) so as to identify the most appropriate cancellation algorithm in a balanced/fair scenario. After settling on a given algorithm, we then evaluate the three setups described so as to understand the capability of the selected SI cancellation algorithm in varying SDR locations/setups. Finally, choosing the SI cancellation algorithm with best performance and the fairest scenario, the effect of employing the SCS-HK scheme to further enhance the effect of SI cancellation is assessed.

### 5.1. Self-Interference Cancellation Algorithms Results

In this section we evaluate the usage of the LMS, NLMS, RLS and QRD-RLS algorithms for cancelling the SI signal in the fairest scenario of Setup I. For that, we also employ passive suppression (see Section 2.4) by means of fixing the polarity of Bob’s transmit antenna to $90^{\circ }$ relative to the other antennas for better SI cancellation as shown in Figure 5, Figure 6 and Figure 7. In all these tests, Alice’s power is fixed at 20 dBm, and Bob’s jamming power varies from 20 dBm to 32 dBm (in increments of 2 dBm).

The setting up of parameters of the SI cancellation algorithms was made according to [34], with the number of filter taps *t* being set to the maximum that the processor could handle considering the maximum sample rate *S* attainable. The parameters used are presented in Table 1, where μ is the step size of LMS and NLMS algoritms [34], and σ and λ are, respectively, the normalization and forgetting factors of the RLS-type algorithms [35].

**Figure 8:**
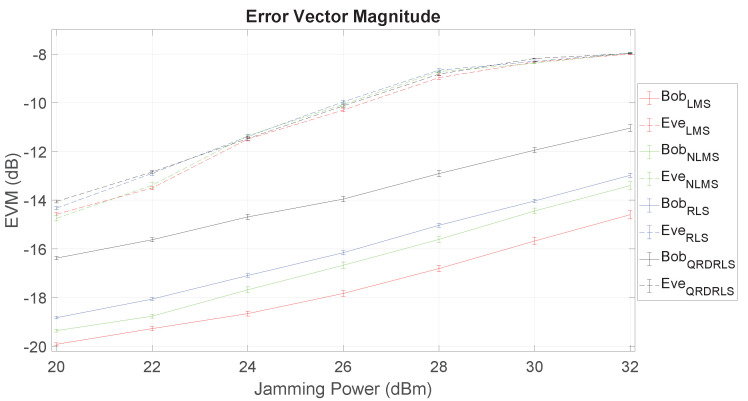
Error vector magnitude (EVM) as function of jamming power for Bob and Eve at Setup 1.

The measured EVM for Bob and Eve as a function of the jamming power is presented in Figure 8. The results show evidence of the advantages of using FD jamming and the ability of Bob to cancel his SI. In fact, it can be observed that Bob experiences a much lower EVM than Eve, and the LMS algorithm largely outperforms all the others. The significant gap between Bob and Eve’s EVM curves means that it is possible to increase the jamming power to degrade Eve with less harm to Bob.

### 5.2. Favorable and Unfavorable Setups Results

With the LMS algorithm established, the effect of the SDRs’ positions are now evaluated by also considering the remaining Setups II and III.

**Figure 9:**
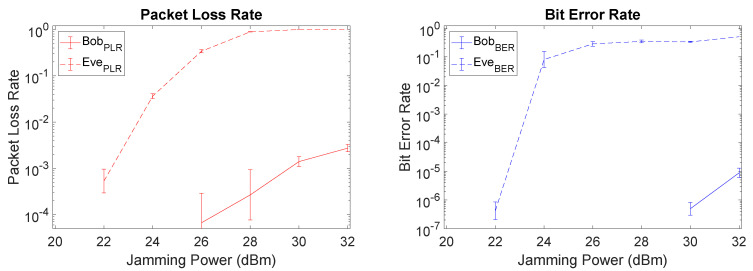
Packet loss rate (PLR) and bit error rate (BER) plots of Setup I (fairest) when using least mean square (LMS).

We start by considering the additional metrics of PLR and BER for Setup I, as depicted in Figure 9. There is a clear gap between Bob and Eve with respect to PLR and BER values, meaning that a relevant security level is attained through SI cancellation mechanisms at Bob. Particularly, at 26 dBm Bob has a very low PLR being capable to obtain almost all packets (PLR = $6.67\times 10^{-5}$), while Eve only recovers nearly 1/3 of the packets (PLR = $3.39\times 10^{-1}$). From those packets recovered by Eve, only about 28% of the bits are received without error, thus severely compromising Eve’s ability to retrieve the original data. Increasing the jamming power, worsens the PLR and BER for Eve up to a certain point, albeit with a corresponding penalty for Bob as well. Note that the BER and PLR values for Bob are omitted when we had a lack of precision in the real-world prototype results.

**Figure 10:**
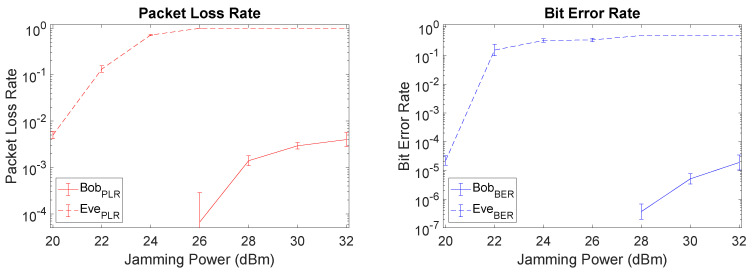
PLR and BER plots of Setup II (favorable for Bob) when using LMS.

When comparing Setup I (fairest) with Setup II (unfavorable to Eve), since the distance from Alice to Bob was not changed the SI cancellation capability of Bob remains roughly the same, as depicted in Figure 10. With Eve at a larger distance from Alice, we note a left shift on Eve’s PLR and BER curves, i.e., Eve experiences higher PLR and BER for lower levels of jamming power. This leads to a larger gap between Bob and Eve which is favorable from a security perspective, but expected due to the unfavorable setup for Eve at a larger distance from Alice.

**Figure 11:**
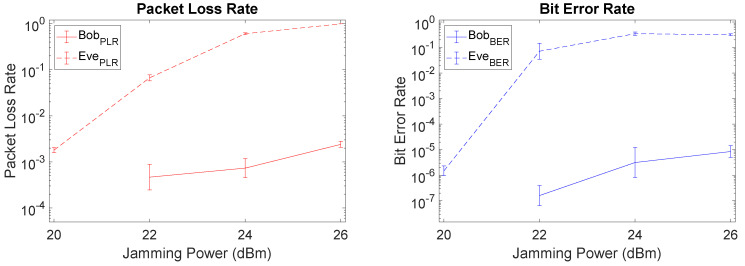
PLR and BER plots of Setup III (favorable for Eve) when using LMS.

With Bob located further away from Alice than Eve (Setup III) the gap between Bob and Eve is reduced due to an increase in PLR for Bob. In this case, increasing the jamming power is not an effective mechanism, since it will also increase Bob’s PLR, as depicted in the left-hand side of Figure 11. However, when packets go through to Bob, somewhat surprisingly Bob’s BER remains low and there is still a large gap between Eve and Bob, as depicted on the right-hand side of Figure 11. This means that it is still possible to increase the jamming power up to some point (altough not as high as in the previous setups) and achieve an acceptable BER level, as long as we are willing to accept a trade-off with the increasing PLR for Bob. For example, for a jamming power of 26 dBm Bob has a PLR of $2.4\times 10^{-3}$, but an acceptable BER of $8.5\times 10^{-6}$, with Eve still being affected in her reception capability through a high PLR of $9.9\times 10^{-1}$ and a high BER of $3.2\times 10^{-1}$.

In summary, this assessment allowed us to perform a realistic prototype evaluation of the effectiveness of SI cancellation mechanisms for security purposes. However, with different trade-offs, these mechanisms have shown to be effective in facilitating a security gap between Bob and Eve by taking advantage of the knowledge of the interference signal on Bob’s side. In particular, be it a favorable or unfavorable scenario, self-interference generation with SI cancellation is adequate to provide a relevant security gap between Bob and Eve. Naturally, this gap narrows when Eve is in an advantageous situation, but even for that case we have shown that it is possible to achieve a prescribed security level, as long as one is willing to accept a penalty in the packet-loss rate.

### 5.3. SCS-HK with Self-Interference Cancellation

In this section we explore the combination of SI cancellation with the scrambled coding for secrecy scheme (SCS-HK) described in Section 2.1 to enhance the security advantage obtained from SI cancellation, as shown in previous sections. To perform this evaluation, the LMS filter is chosen and the fairest scenario is set.

The SCS-HK scheme is a coding for secrecy mechanism that exploits the usage of scrambling with a hidden key and puncturing of that key prior to transmission to increase secrecy in communication. This technique grants reliability to Bob and enhanced confusion to Eve, when Eve’s channel is worse than Bob’s [4,29]. When combined with the jamming component of this work, the goal is to enhance the degradation of the channel between Alice and Eve caused by the jamming to further increase Eve’s BER. For that, we will evaluate the impact of several puncturing patterns (i.e., number of bits punctured in the codeword) for selected values of jamming power (chosen according to the previous results for low PLR and BER). Specifically, we consider a range of 0,20,44,65,85,100 bits punctured, for jamming values of 22,24,26,28 dBm. Note that the case of 0 punctured bits corresponds to the case in which the additive scrambler is employed without erasing the key before transmission, and it is here considered for comparison. In addition, note also that, if Bob doesn’t jam Eve, both have the same SNR (as implied by Setup I) and thus, the SCS-HK technique does not provide any security advantages for Bob against Eve.

**Figure 12:**
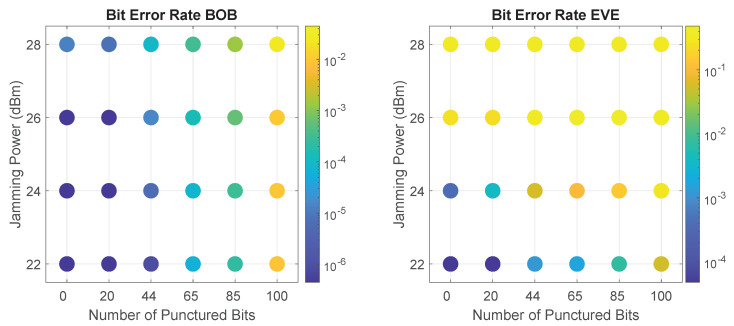
Bob and Eve’s BER performance of SCS-HK combined with full-duplex (FD) jamming as function of the number of bits punctured and Bob’s jamming power. Bob’s results assumes that he employs the LMS technique on the cancellation of SI.

As the SCS-HK affects only the payload’s packet, we will base the analysis only on the BER metric. Figure 12 presents Bob and Eve’s BER results as function of the number of punctured bits and jamming power. Results show the clear impact of an increase in the SCS-HK’s number of punctured bits in the decrease of the BER performance, particularly for Eve given some values of jamming. The main goal is looking for the combinations where Eve’s BER increases quicker than Bob’s BER allowing us to have a combination where puncturing interference is significantly heavier on Eve (because it is combined with jamming and in Bob the jamming is canceled).

The one situation in which increasing the number of puncturing bits produced no effect is when a high jamming power of 28 dBm is employed, which already causes a high BER for Eve. Therefore, there is no interest of using the SCS-HK scheme with this level of jamming power, so it is excluded from further analysis.

In the same way, when considering 22 dBm of jamming power, as this doesn’t degrade Eve’s channel considerably, the SCS-HK scheme not only affects her, but also impacts Bob considerably. Thus, the SCS-HK scheme is not worth implementing in a transmission with this jamming power value in this scenario. Moreover, there are combinations with jamming >22 dBm in which Eve experiences a similar BER, while Bob undergoes a much lower one. For example, for 22 dBm of jamming power and 100 bits punctured, Eve’s BER is $5.60\times 10^{-2}$ and Bob’s BER is $9.61\times 10^{-3}$, while with 24 dBm of jamming power and 44 bits punctured, Eve’s BER is $6.11\times 10^{-1}$ and Bob’s BER is $6.07\times 10^{-6}$, i.e., with relatively the same value of Eve’s BER, Bob’s BER is significantly lower. Therefore, this jamming/puncturing combination is also excluded from further analysis.

When using either 24 dBm or 26 dBm of jamming power, the impact of the SCS-HK scheme is obvious. However, once again, it is not possible to strictly say which combination is the best, since the choices present trade-offs between the packet loss and data errors that we accept on Bob and the received packets and correct data that we allow Eve to collect. The most interesting combinations are addressed below.

Fixing the same order of magnitude on Bob’s BER ($10^{-5}$), it is important to mention that the combination of 26 dBm jamming power and 44 bits punctured significantly outperforms the 24 dBm jamming power with 65 bits punctured, achieving $3.54\times 10^{-1}$ and $1.10\times 10^{-1}$ on Eve’s BER, respectively. This means the SCS-HK scheme and self-interference/jamming are two complementary approaches to increase the security level for wireless networks.

In conclusion, it is possible to secure a communication only using the noise generation; however, when combined with the SCS-HK scheme, for a selected combination of parameters (i.e., number of bits punctured and jamming power) it is possible to increase Eve’s BER with only a minor effect on Bob, thus improving the security level.

## 6. Conclusions

In this paper, we presented a SDR proof-of-concept implementation in GNU Radio of the SCS-HK coding for secrecy scheme combined with FD jamming interference generated by the legitimate receiver against an unknown eavesdropper. Several least squares based algorithms to cancel SI were also implemented and evaluated, with the results showing that the LMS algorithm outperforms the others for the same environment. Results also showed that the jamming technique in isolation grants a clear advantage to the legitimate receiver over an eavesdropper, due to the ability of the first to cancel the SI. We observe the PLR and BER’s gaps increasing to an advantageous situation and decreasing to a disadvantageous one; either way showing that it is possible to secure the communication in each case. Finally, when the jamming technique is combined with the adapted SCS-HK scheme, a security improvement was observed by increasing Eve’s BER with a minor effect on Bob, for several configurations of the jamming power and the number of SCS-HK’s punctured bits.

Future directions of this work include the usage of directional antennas to achieve a larger security gap between Bob and Eve, as well as employing other PLS code variants that may further improve the security gap along with improved recovery from wrong bits, thus improving the throughput.

## Figures and Tables

**Table 1:** SI cancellation algorithm’s parameters.

Algorithm	μ	σ	λ	*t*	*S*
LMS	0.0075	-	-	64	405 KHz
NLMS	0.0075	-	-	32	405 KHz
RLS	-	1	1	12	200 KHz
QRD-RLS	-	1	1	12	200 KHz

## Data Availability

Data available in https://github.com/IT-UniversityCoimbra-SDRWirelessComm/Full-Duplex-Jamming-for-Secrecy.
